# Book Reviews

**Published:** 1871-01

**Authors:** 


### BIBLIOGRAPHICAL NOTICES.

*Pathologie Der Zahne, Mit Besonderer Rucksicht Auf Anatomie Und Physiologie. Bearbeitet Von Prof. Dr. C. Wedl. Leipsig Verlag Von Arthur Felix.*

This valuable work of three hundred and sixty-two pages and one hundred and two well executed wood engravings, has been received, and we find that it contains much valuable information concerning the dental structures.

We trust it may soon be translated into English, so as to place it within the reach of all interested in the subject.

---

*Twelfth Annual Report of the Chicago Eye and Ear Infirmary.* Presented by the Board of Surgeons for the year 1869.

The total number of cases of diseases of the eye treated during the year was 759, and of diseases of the ear 114.

---

*Scientific American.*—Twenty sixth year. This splendid weekly, greatly enlarged and improved, is one of the most useful and interesting journals published. Every number is beautifully printed on fine paper, and elegantly illustrated with original engravings, representing new inventions novelties in mechanics, manufactures, chemistry, photography, architecture, agriculture, engineering science and art.

Farmers, mechanics. inventors, engineers, chemists, manufacturers, and people of all professions and trades will find the Scientific American of great value and interest.

Its practical suggestions will save hundreds of dollars to every household, workshop, and factory in the land, besides affording a continual source of valuable instruction. The editors are assisted by many of the ablest American and European writers, and having access to all the leading scientific and mechanical journals of the world, the columns of the Scientific American are constantly enriched with the choicest information.

An official list of all the patents issued is published weekly. The yearly numbers of the Scientific American make two splendid volumes of nearly one thousand pages, equivalent in size to four thousand ordinary book pages. Specimen copies sent free. Terms—$3 a year; $1.50 half year; clubs of ten copies for one year, at $2.50 each, $25.00, with a splendid premium to the person who form the club, consisting of copy of the celebrated steel plate engraving, "Men of Progress." Published by MUNN & Co. 37 Park Row, N. Y.

---

*The Manufacturer and Builder.*—Published by Western & Co. Park Row, N. Y. is one of the most useful and attractive of this class of magazines, and is profusely illustrated with designs and drawings of machinery [illegible]. etc. Much useful reading matter is found in every number, which will prove interesting to all professions and trades. Terms $1.50 a year.

---

*The Herald of Health.*—Published monthly by Wood & Holbrook, 13 and 15 Laight St. N. Y. Terms $2, a year.

Of this publication the "Scientific American" remarks "it is a journal which contains more sensible articles on subjects of a practical moral bearing, than are to be found in any other monthly that comes to our sanctum." Its articles are the production of sound thinkers and able writers, and render it a work of great value.

---

*Arthur's Lady's Home Magazine.*—The January number has reached us, filled, as usual, with interesting matter, and cannot be excelled by any competitor of its class. The colored steel fashion plate is one of the finest; the rich Cartoon entitled "The Skein Winders is a picture of high artistic beauty. "Grandpa's Darling," "Going to School," and "Coming from School," are three charming pictures. In elegance and beauty and attractive reading, this Magazine will always prove interesting to patients in a dental office, as well as in the home circle. Published by T. S. Arthur & Sons, Philadelphia, Pa. Terms $2. a year. Three copies for $5.

---

*The Children's Hour*, with its wealth of sweet pictures and attractive reading matter, cannot fail to be a welcome guest to all the little ones of a household. Its pure and benign influence will be appreciated by every parent who supplies his children with it. Published by T. S. Arthur & Sons, Philadelphia. Terms $1.25 a year.

---

*The Bright Side* is the title of an interesting child's paper, of eight pages, which is published weekly and semi-monthly. It contains serial and short stories, poetry, topics of the times, news, scientific articles, biographical sketches, historical articles, stories of travel and adventure, bright side sermons and illustrations appropriate to the text and the times. The semi-monthly edition is intended especially for Sunday Schools. Address Bright Side Company, Chicago Ill. Terms $1.00 a year for the weekly edition.

---

*The American Agriculturist*, for the Farm, Garden and Household. Orange Judd & Co., 545 Broadway, N. Y. Terms $1.50 a year. This standard work has now reached its thirtieth volume, and is so well known, that it requires no words of ours to enlighten the public concerning its merits. We can but wonder how its Publishers can supply so elegant a magazine for the subscription price, as every number is profusely illustrated, printed on fine paper and presents a very attractive appearance. It has become a welcome guest to every farm house where it is received, as it contains matter interesting to the young as well as to the old.

---

*Scribner's Monthly.*—Scribner & Co., 654 Broadway, N. Y. Conducted by L. G. Holland. Terms $3 a year. The January number of this popular illustrated monthly magazine has been received, and the following is the table of contents: Fairmount Park, illustrated. Kings of the Air, illustrated. The Goblins of the Ice, illustrated. Tartini's Dream Music. The Christmas Door, illustrated. Mirabel's Christmas. Natasqua. Lucky Peer, illustrated. Terms of Peace. How we escaped war with Spain. Ships, illustrated. The Flight of the birds. The Northern Lights, illustrated. Strasburg after the Surrender, illustrated. Wilfrid Cumbermede, illustrated. My Wild Sis. A Christmas Eve in Germany. Topics of the Times. The Old Cabinet. Home and Society. Books and Authors at Home. Etchings, etc. Christmas Carol, making it an attractive and interesting work. The articles are from the pens of some of the best authors of our times.

---

*The New York Observer Year Book and Almanac*, 1871.–This valuable work has been received and contains useful information on almost every subject. It is divided into Astronomical, Civil and Commercial, Ecclesiastical, Church Work, Educational, Agricultural and Miscellaneous Departments. Price $1.00. All subscribers to the *New York Observer* will receive this work gratuitously, upon paying their subscriptions for one year, in advance.

The first Directory of New York, published in 1786, is reprinted entire in this work. It is a very rare book, and four years ago a copy of it brought $100 at public auction.

*The Art Review.*—A Record of Art-Progress in America. E. H. Trafton, Publisher, 39 Park Row, N. Y., and 115 Madison St., Chicago. The January number contains the following illustrations: Under the Daisies, a full page etching, by True Williams. Chicago Statuary. Chicago Academy of Design Building. A Hint to Artists, Comic Cartoon, by F. S. Church. Poems and Prose, and other interesting art-matter. The Art-Review is a bi-monthly. Terms $1.50 a year.

